# Refixation of the anterior cruciate ligament: A biomechanical analysis of suture techniques in a porcine model

**DOI:** 10.1002/jeo2.12011

**Published:** 2024-03-15

**Authors:** Christoph Lutter, Natalie Hiller, Jan‐Oliver Sass, Jessica Hembus, Gareth Jones, Danny Vogel, Justus Groß, Rainer Bader, Thomas Tischer

**Affiliations:** ^1^ Department of Orthopaedics Rostock University Medical Center Rostock Germany; ^2^ School of Clinical and Applied Sciences Leeds Beckett University Leeds UK; ^3^ Department for General, Visceral, Thoracic, Vascular and Transplantation Surgery University Hospital Rostock Rostock Germany

**Keywords:** ACL, experimental biomechanics, refixation, rupture, suture technique

## Abstract

**Purpose:**

Refixation of acute anterior cruciate ligament (ACL) tears represents an increasingly popular treatment option. Systematic evaluations of various suture technique parameters are still pending. We therefore aimed to evaluate the mechanical pull‐out outcomes of various suture methods for optimization of ACL refixation.

**Methods:**

Sixty fresh knees from mature domestic pigs were dissected and the femoral attachment of the ACL was peeled off. The 60 knees were divided in 10 groups and sutured as follows: (A) one suture (1, 2, 4 and 6 passes), (B) two sutures (2, 4 and 6 passes each; sutures knotted together as a loop) and (C) two sutures (2, 4 and 6 passes each, sutures knotted separately). The pull‐out test was conducted using a validated electrodynamic testing machine. First occurrence of failure, maximum pull‐out load and stiffness were measured. Suture failure was defined as pull‐out of the ACL.

**Results:**

Two‐point fixation, using two sutures, with at least two passes, showed the most favourable biomechanical stability. The maximum pull‐out load was significantly higher with two sutures (529.5 N) used compared to one (310.4 N), *p* < 0.001. No significant differences were found for maximum pull‐out loads between two‐point fixation versus one‐point fixation but stiffness was significantly higher with two‐point fixation (107.4 N/mm vs. 79.4 N/mm, *p* < 0.001). More passes resulted in higher maximum pull‐out loads.

**Conclusion:**

The results suggest using two independent sutures, refixed separately and at least two suture passes, is appropriate for ACL refixation. More suture passes provide additional strength but are technically challenging to achieve during surgery.

**Level of Evidence:**

Level IV.

**Abbreviations**
ACL: acute anterior cruciate ligament
CL: cinch loop

## INTRODUCTION

Anterior cruciate ligament (ACL) reconstruction has provided excellent clinical results and for a variety of patient groups [11, 15, 16]. However, donor site morbidity, loss of proprioception with impaired neuromuscular function and failure rates of 8%–25% must be considered when replacing the anatomical structure [19, 20, 21]. In addition, tunnel placement, graft fixation and revascularization/ligamentization are key surgical factors [19, 20, 21]. In ACL reserving techniques, the (partially) ruptured ligament is sutured or reattached and thereby preserved. The benefits of arthroscopic ACL preserving techniques to restore normal knee function have been described and widely discussed in recent years [10, 11, 12, 17]. Indications for these techniques, in particular cases with a femoral avulsion of the cruciate ligament, are described in the literature and should be considered by surgeons as a viable therapeutic option [10, 12]. The prerequisite for these techniques appears to be a tear close to the proximal attachment of the cruciate ligament and good tissue quality of the ACL stump [10]. In contrast to isolated ACL injuries, ACL preservation also gains new importance in the context of multiligamentous injuries of the knee [6]. In severe knee injuries, it is common for multiple ligamentous damage to be addressed immediately to restore joint stability and reduce subsequent damage. Since several torn ligaments may require multiple grafts, their sparing use is advisable and early surgical ACL preservation seems reasonable [6]. The biomechanical properties of the ACL refixation is crucial to healing, rehabilitation and functional outcome. As such a stable pull‐out resistant suture technique is essential. Various suture techniques in regard to remnant‐preserving and retensioning have been trialled, including single‐, loop‐ and triple‐stitches (single‐stitch: passing one suture through the remnant. Loop‐stitch: passing one suture loop through the remnant, free ends of suture retrieved through the loop. Triple‐stitch: passing three sutures through the remnant; medial to lateral, anterior to posterior and medial to lateral) [5, 14]. In their recent biomechanical comparison study, Ryu et al. [14] reported superior pull‐out strength of the loop‐ and triple‐stitch as compared to the single stitch in ACL remnant preserving and re‐tensioning reconstruction. However, a systematic biomechanical evaluations of suture technique parameters including the number and passes of the suture, as well as the knotting technique used is warranted. Clinical studies predominantly refer to the cinch loop (CL) techniques or modified Bunnell‐type stitch configurations [1, 2]. In contrast, bony fixation techniques such the knotted cortical button fixation have recently been biomechanically evaluated for stability and found to have superior peak loads and reduced gap formation compared with all other groups [1, 2].

The principle aim of the study was to evaluate the mechanical pull‐out behaviour of various suture methods for optimization of ACL refixation using a porcine model. We hypothesized that two sutures and increased number of passes through the ACL would result in higher pull‐out forces and stiffness.

## MATERIALS AND METHODS

### Preparation of specimen

**Figure 1:**
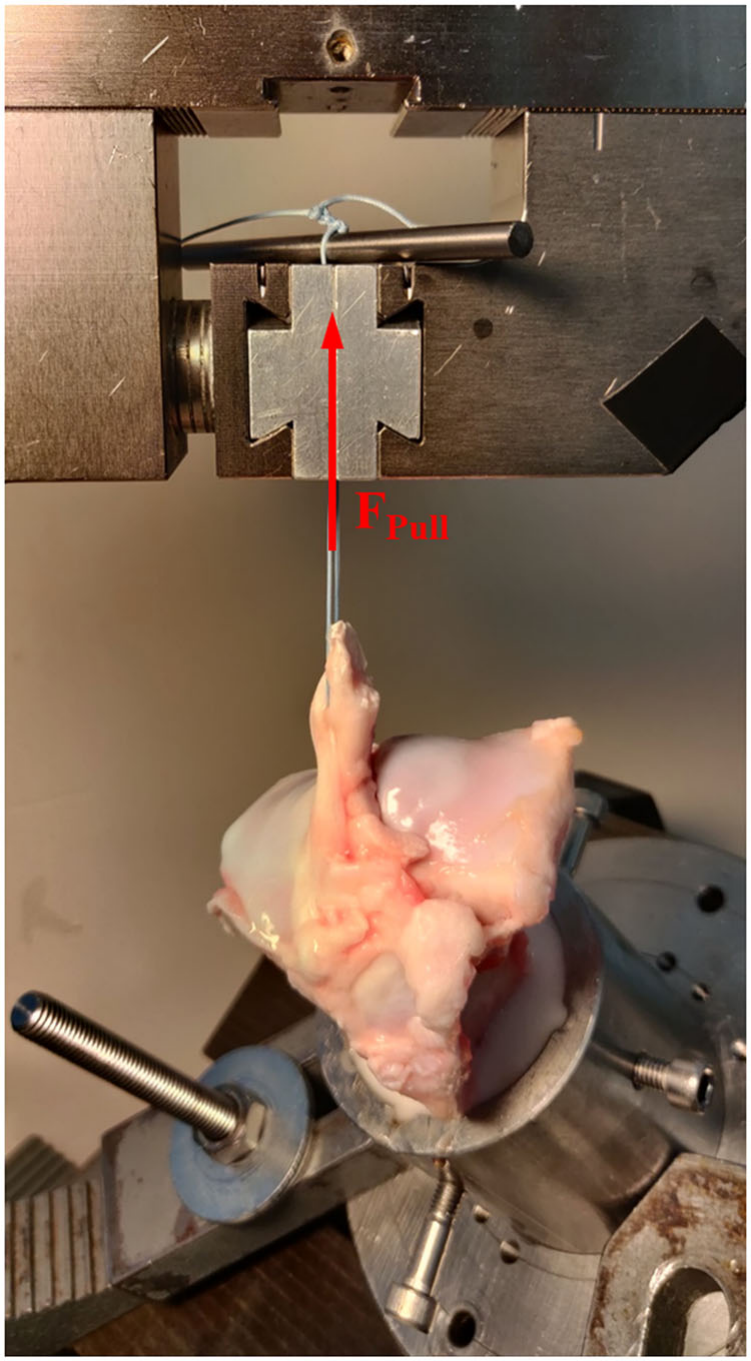
Test setup with one suture guided through clamp and a steel pin and indication of the force direction and reference length L_0_.

Sixty fresh knees from mature domestic pigs were dissected and the femoral attachment of the ACL was peeled off. During the following steps, the structures to be tested were kept moist at all times with a sodium chloride solution. Subsequently, the proximal tibia was embedded in a test cassette using epoxy resin (RenCast® FC 52/53 Isocyanat/FC 53 Polyol; Huntsman Advanced Materials GmbH). The embedded proximal tibia was transferred to the test setup and the ACL was then sutured using the refixation techniques described below. Subsequently, the tilting table of the test setup was adjusted to 30° flexion so that the direction of the axial load was aligned with the longitudinal axis of the prepared tendon (Figure 1).

### ACL refixation techniques

**Figure 2:**
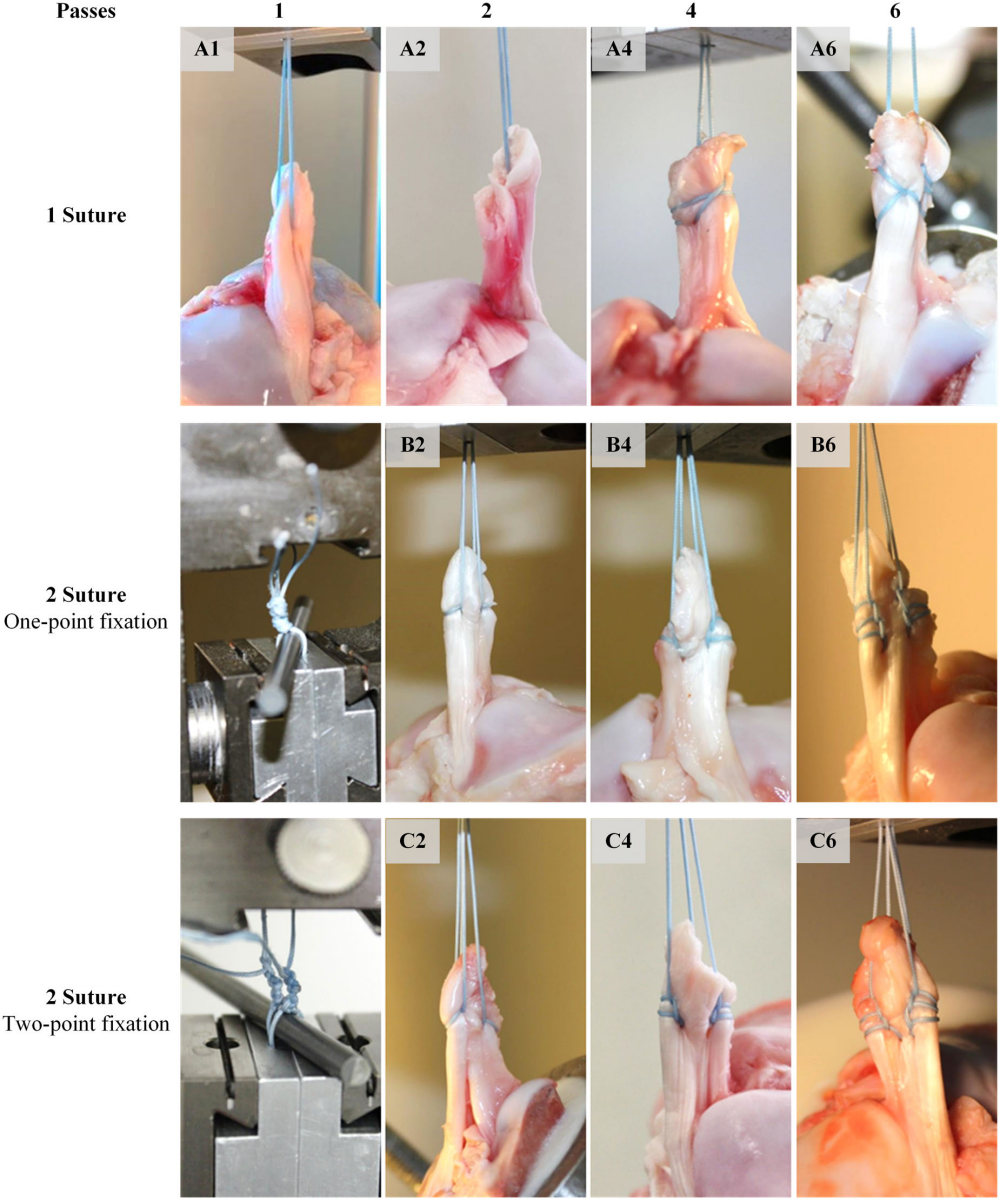
Sixty knees were divided in 10 groups: (A) 1 suture (A1: 1, A2: 2, A4: 4, A6: 6 passes each), (B) 2 sutures (B2: 2, B4: 4, B6: 6 passes each; one‐point fixation) and (C) 2 sutures (C2: 2, C4: 4, C6: 6 passes each; two‐point fixation).

The 60 knees were divided in 10 groups with each *n* = 6 specimens: depending on the number of sutures used (one or two) and the number of passes of each suture through the tissue (1, 2, 4 or 6 passes). The groups are demonstrated in Figure 2 as: (A) 1 suture (A1: 1, A2: 2, A4: 4, and A6: 6 passes each); see Figure 2 top row. (B) 2 sutures (B2: 2, B4: 4, and B6: 6 passes each; one‐point fixation); see Figure 2 middle row. (C) 2 sutures (C2: 2, C4: 4, and C6: 6 passes each; two‐point fixation); see Figure 2 bottom row. All ACL repairs were performed by a board‐certified orthopaedic surgeon with specialisation in sports orthopaedics. A Scorpion‐Needle® (Arthrex) was used for all stitches through the ACL and a FiberWire® #2 (Arthrex) for all sutures. Modified loop stitches were used where more than one pass was made through the ACL. The stitches were placed in such a way that the sutures were supported by the adjacent loop. One‐point fixation and two‐point fixation was simulated by either knotting sutures together as a loop (one‐point fixation) or separately (two‐point fixation) over the pulley of the testing device.

### Suture fixation in testing device

For simulated femoral fixation of the sutures, a clamp with a central cut‐out was used. The sutures were guided through the clamp of the test rig in a manner that prevented them from being trapped. The deflection was performed on a ∅5 mm steel pin. The free ends of the nodes were marked to detect the setting of the knots after biomechanical testing.

### Biomechanical testing and data acquisition

All iterations were performed using an electrodynamic testing machine (LTM 5; ZwickRoell GmbH & Co. KG). The programme testXpert R (v.1.8.1., ZwickRoell GmbH & Co. KG) was used for the supervision of the test and the data acquisition. The mechanical loading was performed in accordance with the procedure described by Bachmaier et al. [1]. After mounting the specimens in the testing machine, pretensioning was initially performed to generate reproducible conditions for all tests. The load axis was aligned with the suture to simulate a worst‐case scenario and to prevent stress peaks at the deflection points of the suture material.

The biomechanical test started with preloading of 60 N (cross‐head speed of 0.5 mm/s), where the displacement sensor was set to zero. Initial preconditioning was informed by Bachmaier et al. [1] commencing with 10 cycles at 0.5 Hz between −3 mm and 0 mm to simulate intraoperative motion between full extension and 90° flexion. A preload of 60 N was then applied, and the displacement sensor was set back to zero. In the second part of the preconditioning, eight phases with 500 cycles each were performed, where a sinusoidal tensile load at 0.75 Hz was applied. The minimum displacement was 0 mm and the maximum increased from 1 to 8 mm in 1 mm increments. Accordingly, each sample was loaded for 4000 cycles. The cyclic preconditioning was followed by a pull‐to‐failure test with a cross‐head speed of 50 mm/min.

**Figure 3:**
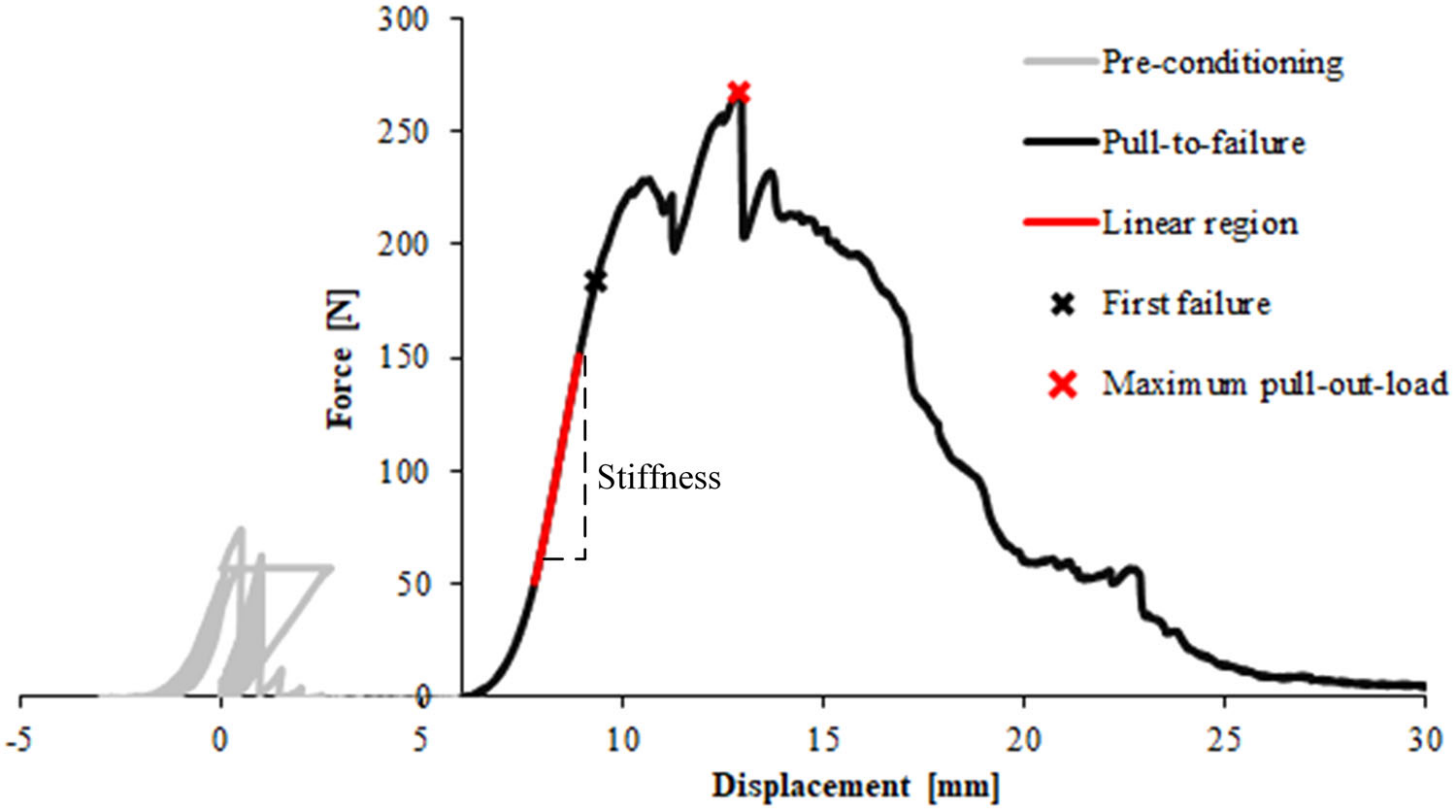
Force‐displacement curve of a sample of group C1 indicating the relevant biomechanical parameters for evaluation of the different ACL repair techniques.

Data was collected over the entire duration of the test and checked for irregularities. The load‐displacement curve of the pull‐to‐failure test was used to determine the stiffness, the force at first occurrence of failure, and the maximum pull‐out‐load (Figure 3). The maximum pull‐out‐load represents the highest force that the suture‐remnant bond can withstand before it fails completely. The stiffness (in N/mm) was defined as the slope of the linear regression of the first linear region of the force‐displacement curve. The regression was evaluated in the same force range for each group. The first failure (in N) was defined as the 0.2% strain offset of the linear region, where the reference length L_0_ was measured between the distal insertion site of the ACL on the tibia up to the suture. First failure represents the transition from the linear‐elastic to the plastic range in the diagram, that is the range in which the applied load is no longer reversible. This occurs as soon as the first fibres in the bundle break (Figure 3).

### Statistical analysis

Microsoft Excel (Version 16.47; Microsoft Corp.) was used for data collection; statistical analyses were performed with SigmaStat software (Systat Software Inc; Version 11.0). Values were checked for normal distribution using the Shapiro–Wilk test. To determine the difference among groups, a *t* test or rank sum test was used. A nonparametric Kruskal–Wallis one‐way analysis of variance on ranks was used for nonnormally distributed data among several groups. Unless otherwise stated, data are expressed as mean, range and standard deviation. A *p *< 0.05 were considered statistically significant.

## RESULTS

The force‐displacement curve characteristics during the pull‐out‐tests were similar for all groups. A linear region was observed followed by either a sharp drop in force, or a decreased slope, which corresponded to the first failure of the ACL repair. As gradual pull‐out of the suture occurred, multiple increases and drops in the force‐displacement curve occur.

Single suture pass (group A1, 1 suture, 1 pass) showed insufficient stability and suture failure (suture pull‐out of ACL) during preconditioning in all cases and therefore no biomechanical parameters were calculated.

### Failure mode

Suture failure (suture pull‐out of ACL) occurred as follows in the various groups: (A) A2: four sutures were pulled out of the ACL remnant. In A4 also four suture failures occurred while in group A6 only one suture failure was detected. (B) In B2 and in B4 six suture pull‐outs were detected each while in B6 only four suture failures were found. (C) C2: six sutures were pulled out of the ACL remnant. In C4 three suture failures occurred while in C6 only one suture failure was detected. Suture material failure (rupture of suture) occurred in all other cases.

### Biomechanical parameters

**Figure 4:**
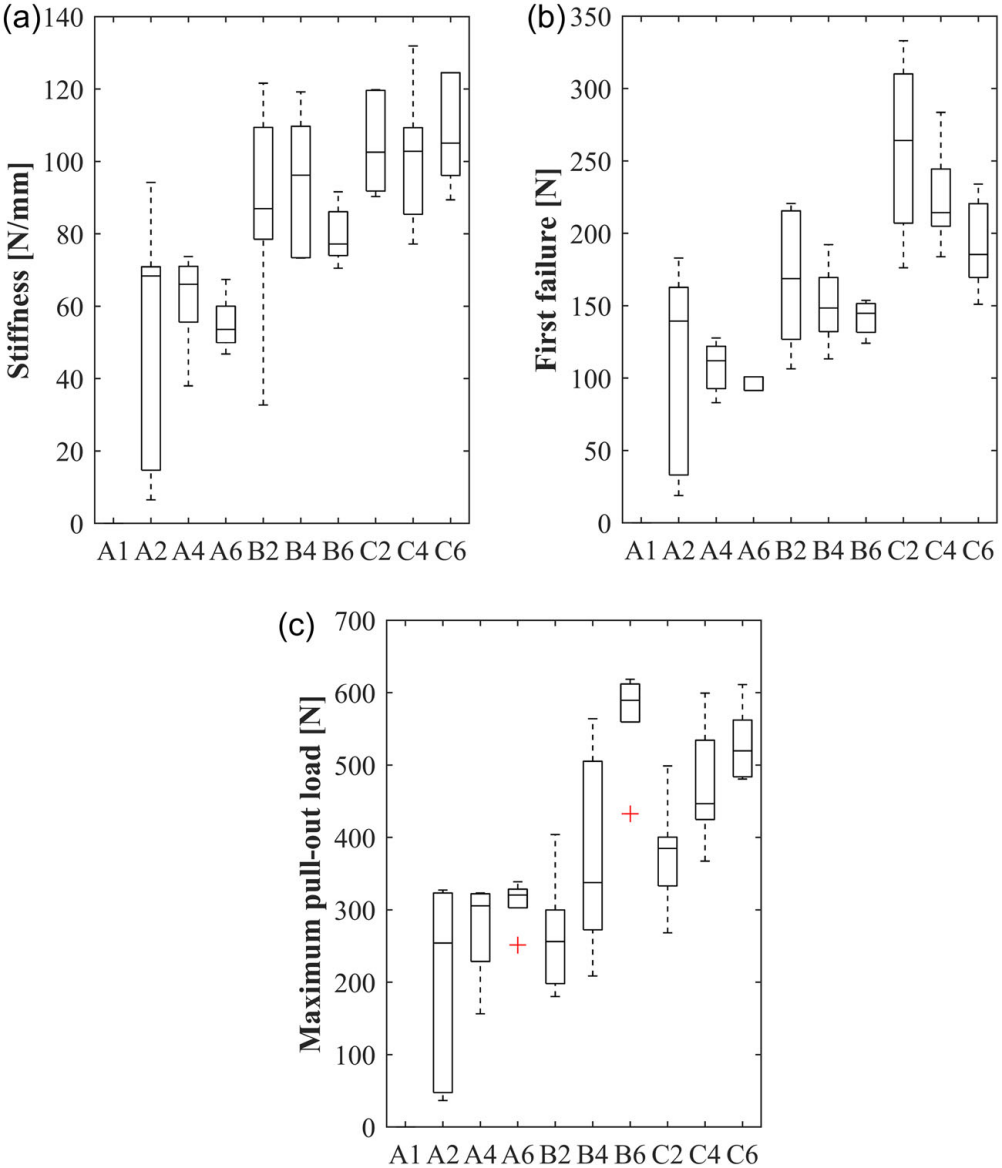
Boxplots of the (a) stiffness in N/mm, (b) force at first failure in N, and (c) maximum pull‐out‐load of the groups (A): 1 suture (A1: 1, A2: 2, A4: 4, A6: 6 passes), (B): 2 sutures (B2: 2, B4: 4, B6: 6 passes each; one‐point fixation), and (C) 2 sutures (C2: 2, C4: 4, C6: 6 passes each; two‐point fixation).

Results of the measured stiffness, force at first occurrence of failure and maximum pull‐out‐load are summarized in Table 1 and Figure 4. Stiffness increased from group A to B and C, with a significant difference between A and C (*p* = 0.038). For the different ACL repair techniques, the force at first occurrence of failure and the maximum pull‐out‐load tended to increase from A to B and C. The number of passes within a group had no effect on stiffness. Within the groups, the number of passes had no effect on the force at first occurrence of failure. However, the maximum pull‐out load was significantly influenced by the number of passes, with two passes always resulting in the lowest values and six passes resulting in the highest values within the group. When all tests were appraised, only group C was found to be significantly better than group A (Table 1, Figure 4).

**Table 1:** Stiffness, first failure and maximum pull‐out load of the groups A, B, and C.

	Group A	Group B	Group C	
Suture technique	Single‐suture	One‐point fixation (sutures knotted together as a loop)	Two‐point fixation (sutures knotted separately)	
Stiffness [N/mm]				
1‐pass^a^	‐	‐	‐	
2‐passes	63.5 ± 26.2	86.0 ± 30.9	104.4 ± 14.4	*p* = 0.038^b^
4‐passes	61.7 ± 13.5	94.7 ± 20	101.6 ± 19.4	*p* = 0.002^b^; *p* = 0.006^c^
6‐passes	55.2 ± 7.4	79.4 ± 7	107.4 ± 15.1	*p* < 0.001^b^; *p* < 0.001^c^; *p* < 0.001^d^
	n.s.	n.s.	n.s.	
First failure [N]				
1‐pass^a^	‐	‐	‐	
2‐passes	110.5 ± 73.4	167.8 ± 48.2	247.6 ± 63.8	*p* = 0.006^b^
4‐passes	108.2 ± 17.8	150.6 ± 28.9	224.2 ± 35.1	*p* < 0.001^b^; *p* = 0.02^c^; *p* < 0.001^d^
6‐passes	91.5 ± 4.5	144.8 ± 29.6	185.4 ± 11.2	*p* = 0.005^b^
	n.s.	n.s.	n.s.	
Maximum pull‐out load [N]				
1‐pass^a^	‐	‐	‐	
2‐passes	207.2 ± 135.8	265.8 ± 80.7	378.4 ± 76.6	*p* = 0.031^b^
4‐passes	273.7 ± 67.3	370.9 ± 140	469.8 ± 83.9	*p* = 0.016^b^
6‐passes	310.4 ± 31.6	566.9 ± 68.9	529.5 ± 51.5	*p* < .001^b^; *p* < .001^c^
	n.s.	*p* = 0.001	*p* = 0.008	

^a^ Failure of all samples during precondition.

b Two‐sutures knotted separately (two‐point fixation) vs. single‐suture.

^c^ Two‐sutures knotted together as a loop (one‐point fixation) vs. single‐suture.

^d^ Two‐sutures knotted separately (two‐point fixation) vs. two‐sutures knotted together as a loop (one‐point fixation).

## DISCUSSION

We found that the failure strength was significantly higher with two sutures as compared to one suture and that suture fixation technique (one‐ or two‐point fixation) does not show a statistically significant difference. Six passes through the ACL revealed the highest ultimate load of failure, while probably for sufficient failure load at least two suture passes are necessary. Stiffness analysis showed results differing from each other for the one‐point and two‐point fixation. Therefore, our hypothesis was found to be correct regarding stability but incorrect regarding the stiffness of the ACL.

We found two‐point fixation showed superior biomechanical stability to one‐point fixation. If suture anchors are used in the knee, the pull‐out force requirements of these anchors must also be considered. Barber and colleagues demonstrated the pull‐out forces of thread anchors to range between 200 and 600 N [3, 4]. Rossleinbroich and colleagues demonstrated pull‐out forces for knotless anchors (PushLock Arthrex) at the knee joint of approx. 200 N [13]. While the suture technique with six passes achieves the best results with regards to pull‐out strength, this method is technically demanding. It becomes even more difficult when two sutures are used (double suture techniques with six passes). Using two sutures with six passes each is challenging to perform arthroscopically and increases the potential risk of damaging the ‘already passed’ sutures with the needle. The intraoperative procedure becomes more complex and technically demanding as the number of sutures and stitches increases. From a biomechanical study it can be deducted that in the native ACL up to 250 N act on the knee [8] so that probably from a practical point of view two sutures with two passes each should be sufficient. In case of bad tendon tissue or more demanding environment, additional passes can increase stability further [8].

While the last few years have been marked by various trends towards ACL preservation, most systems and surgical techniques have failed to gain acceptance. With the follow‐up results getting longer and longer, the inferior results of some ACL preservation techniques are only further confirmed, which is why these surgical techniques tend to decline again and are used less frequently, especially in patients with high activity levels [7]. In the authors' opinion, ACL preservation remains a good surgical option in cases of multiligament injury [6, 7]. The key advantages are the avoidance of tendon‐graft harvesting which could then be reserved for other ligamentous reconstruction, the preservation of proprioception and the less invasive nature of the procedure [6].

In the literature commonly reported ACL refixation methods include the cinch loop (CL) techniques or modified Bunnell‐type stitch configurations [10, 11]. In contrast, bony fixation techniques such as knotless suture anchor and knotted cortical button fixation have recently been biomechanically evaluated for stability and are both used in daily clinical practice [10, 11, 12, 13]. A recent biomechanical comparison study of three different retensioning methods using a porcine model by Ryu and colleagues found that the number and arrangement of stitches through the ACL has a significant effect on the pull‐out force and stability [14]. Although this study was not about the sole preservation or refixation of the original ACL, but about remnant preserving and re‐tensioning reconstruction, it is nevertheless an indication of which suture technique can and should be applied to the ACL. While suturing techniques differ substantially and, our results are nevertheless in agreement with those of Ryu and colleagues, who also demonstrated greater stability for more stitches [14].

Based on the recommendations to use two sutures, the question arises as to how they should be attached to the bone. The choice is between one‐point and two‐point fixation. The presented results suggest that two‐point fixation has the greatest maximum pull‐out force. However, this was not found to be statistically significant. When knotting on a button, this can be easily implemented (e.g., transosseous drilling and separate knotting of the sutures on a cortical button). Alternatively, two anchors could be placed in the bone. Of note, single‐point fixation appears to be superior to two‐point fixation in terms of first failure (initial loosening of the sutures); akin to a pulley effect, by increasing the inherent mechanical advantage and distributing force more equally to all sutures (Figure 2). Which of the two knotting techniques to use is the decision of the surgeon and the intraoperative situation and positioning of the sutures in the ACL.

### Limitations

As a vitro biomechanical study factors such as ligamentous or meniscal co‐stabilization were not considered. Porcine tissue was used and thus only partially reflects the reality in human tissue. However, anatomical and biomechanical studies have shown great similarities between pigs and human ACL and are preliminary used as xenotransplantation in humans [9, 18, 22]. Further, ACLs were detached from the femoral insertion using a scalpel, which does not represent rupture mechanisms in real life, potentially affecting the results. Only one type of suture material was used, and results may therefore vary depending on the suture material. Finally, mechanical load was performed at 30° flexion exclusively, however, this does not represent the full range of motion including rotations, pivoting and axial tensile load during real‐life activities of affected patients.

### Conclusion

In conclusion we suggest using two independent sutures, refixed separately and at least three suture passes, is appropriate for ACL refixation. Although more suture passes provide additional strength they are technically challenging to achieve during surgery and likely unwarranted as it remains unclear whether maximum failure loads in patient populations can exceed these.

## AUTHOR CONTRIBUTIONS

All authors made substantial contributions to conception and design, and/or acquisition of data, and/or analysis and interpretation of data, participated in drafting the article and revising it critically for important intellectual content, approved the submitted version and agreed to be personally accountable for the author's own contributions and ensured that questions related to the accuracy or integrity of any part of the work, even ones in which the author was not personally involved, were appropriately investigated, resolved and the resolution documented in the literature. All authors have read and agreed to the published version of the manuscript.

## CONFLICT OF INTEREST STATEMENT

The authors declare no conflict of interest.

## ETHICS STATEMENT

Ethics approval for the study was granted by the local ethics committee of the Rostock University Medical Centre (A2020‐0098) and data protection requirements were observed.
